# High neutrophil-to-lymphocyte ratio is associated with cancer therapy-related cardiovascular toxicity in high-risk cancer patients under immune checkpoint inhibitor therapy

**DOI:** 10.1007/s00392-023-02327-9

**Published:** 2023-11-13

**Authors:** Elias Haj-Yehia, Raluca I. Mincu, Sebastian Korste, Lena Lampe, Simone M. Margraf, Lars Michel, Amir A. Mahabadi, Péter Ferdinandy, Tienush Rassaf, Matthias Totzeck

**Affiliations:** 1 Department of Cardiology and Vascular Medicine, West German Heart and Vascular Center Essen, University Hospital Essen, Essen, Germany; 2 Department of Pharmacology and Pharmacotherapy, Semmelweis University, Budapest, Hungary; 3 Pharmahungary Group, Szeged, Hungary

**Keywords:** Immune checkpoint inhibitor, Cancer therapy-related cardiovascular toxicity, Neutrophil-to-lymphocyte ratio, Cardiovascular disease

## Abstract

**Background:**

Cancer therapy-related cardiovascular toxicity (CTR-CVT) from immune checkpoint inhibitor (ICI) therapy is still incompletely characterized, and patients with pre-existing cardiovascular disease represent a particularly high-risk cohort. Valid parameters for risk stratification of these patients are missing. Neutrophil-to-lymphocyte ratio (NLR) has been shown to predict mortality and adverse events in other cardiovascular cohorts. The present study aims to examine the predictive capacity of NLR for risk stratification of patients particularly vulnerable for CTR-CVT under ICI therapy.

**Methods:**

We performed an analysis of 88 cancer patients (69 ± 11 years, 25% female) with pre-existing cardiovascular disease under ICI therapy from the prospective Essen Cardio-Oncology Registry (ECoR). NLR was assessed at patient enrollment and the population was divided through receiver operator characteristic (ROC) curve analysis in patients with low (< 4.57) and high (≥ 4.57) NLR. Endpoint was the whole spectrum of CTR-CVT, according to the European guidelines on cardio-oncology. The median follow-up was 357 days (interquartile range (IQR): 150–509 days).

**Results:**

We observed 4 cases of myocarditis, 17 cases of vascular toxicity, 3 cases of arterial hypertension, 22 cases of arrhythmia or QTc prolongation and 17 cases of cardiovascular dysfunction. NLR was associated with overall CTR-CVT by univariable Cox regression (hazard ratio (HR): 1.443; 95% confidence interval (CI) 1.082–1.925; *p* = 0.013). However, this association was attenuated after adjusting for further confounders.

**Conclusion:**

NLR is moderately associated with CTR-CVT in cancer patients with pre-existing cardiovascular disease under ICI therapy. Surveillance of NLR during ICI therapy might be an effective and economically biomarker for risk stratification in these high-risk patients.

**Graphical abstract:**

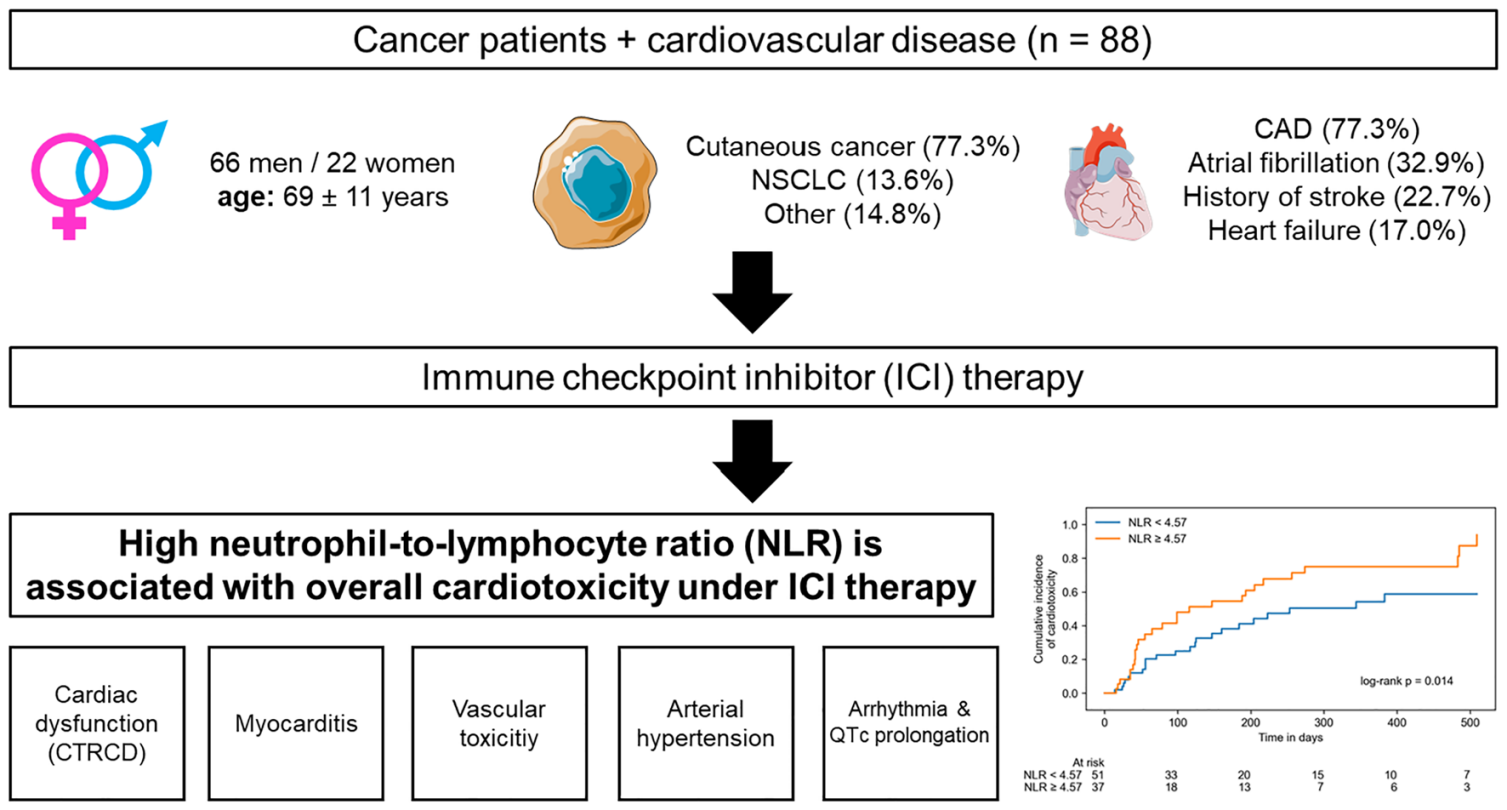

**Supplementary Information:**

The online version contains supplementary material available at 10.1007/s00392-023-02327-9.

## Introduction

Survival of cancer patients has significantly improved over the last decade due to introduction of immune checkpoint inhibitor (ICI) therapy [1–6]. Among others, these treatments are used in the therapy of skin cancer and non-small-cell lung cancer [7–11]. Therapy targets are receptors on the T cell membrane and their ligands on the tumor surface, which allow the cancer cells to be falsely recognized as self-structures and enable them to evade the immune system [12, 13]. ICI therapy blocks this interaction and facilitates their elimination by immune cells [13]. Important immune checkpoint classes approved for ICI therapy so far are programmed cell death protein 1 (PD-1), programmed cell death 1 ligand-1 (PDL-1) and cytotoxic T lymphocyte-associated antigen-4 (CTLA-4). PD-1 is targeted by nivolumab, pembrolizumab, cemiplimab and spartalizumab [13]. Avelumab, durvalumab and atezolizumab inhibit binding of T cells to PDL-1 on the tumor surface and ipilimumab, among others, targets cytotoxic T lymphocyte-associated antigen-4 (CTLA-4) [13].

These therapies come along with cardiotoxic side effects in treated patients [14–16]. These treatment-related effects gained more significance due to an extension of approval in many more cancer entities with tremendous beneficial impact on morbidity and quality of life [15]. The 2022 European guidelines on cardio-oncology and the International cardio-oncology society (IC-OS) recently defined several entities of cancer therapy-related cardiovascular toxicity (CTR-CVT) [17, 18]. One aspect is cancer therapy-related cardiovascular dysfunction (CTRCD), ranging from an increase in cardiac biomarkers or decline in left ventricular contractility to decompensated heart failure. Patients can develop myocarditis or vascular toxicity, appearing as coronary, peripheral and cerebral artery disease or thromboembolic events like myocardial infarction or stroke. Further aspects are new onset of arterial hypertension and QTc prolongation as well as atrial and ventricular arrhythmia. Especially patients with pre-existing cardiovascular disease are endangered to develop CTR-CVT under ICI therapy [17, 19, 20]. Indeed, “hidden cardiotoxicity” of several drugs may manifest only in the diseased heart, i.e., in the presence of cardiovascular comorbidities and their medications [21]. Therefore optimal risk stratification is essential for reasonable therapy decision in these patients.

An important aspect of risk stratification are cardiovascular biomarkers. Among others, troponin, brain natriuretic peptide (BNP) and NT-terminal proBNP (NT-proBNP) were examined in cancer patients under ICI therapy showing an association with CTR-CVT [17, 22]. As cardiovascular side effects from ICI therapy are mainly mediated by increased inflammatory activity, markers for inflammation may be appropriate for risk stratification [23, 24]. One of those markers is the neutrophil-to-lymphocyte ratio (NLR), easily calculated using absolute cell counts of neutrophils and lymphocytes. This parameter gained recent attention as an inflammatory prognostic marker for mortality and cardiovascular events in patients after COVID-19 infection [25, 26]. Furthermore, it is a predictor for short- and long-term mortality in patients with acute coronary syndromes or heart failure [27–30]. In cancer patients, high NLR values are associated with increased mortality [31]. In the context of CTR-CVT, some studies investigated the association with NLR. Cancer patients with ICI-related CTR-CVT showed higher NLR values, but no specific analysis of its predictive capacity for patients with pre-existing cardiovascular disease was performed [32]. In patients with breast cancer under anthracycline therapy, high NLR values were associated with early signs of CTR-CVT [33]. Increased NLR was associated with major adverse cardiac events (MACE) in patients with ICI myocarditis [34]. There are no studies so far, which examined the prognostic capacity of inflammatory biomarkers for cancer patients with pre-existing cardiovascular diseases. As these patients are at a higher risk to develop CTR-CVT under ICI therapy, the aim of this study was to evaluate a possible prediction of NLR for CTR-CVT in this high-risk population.

## Methods

### Study population

**Fig. 1:**
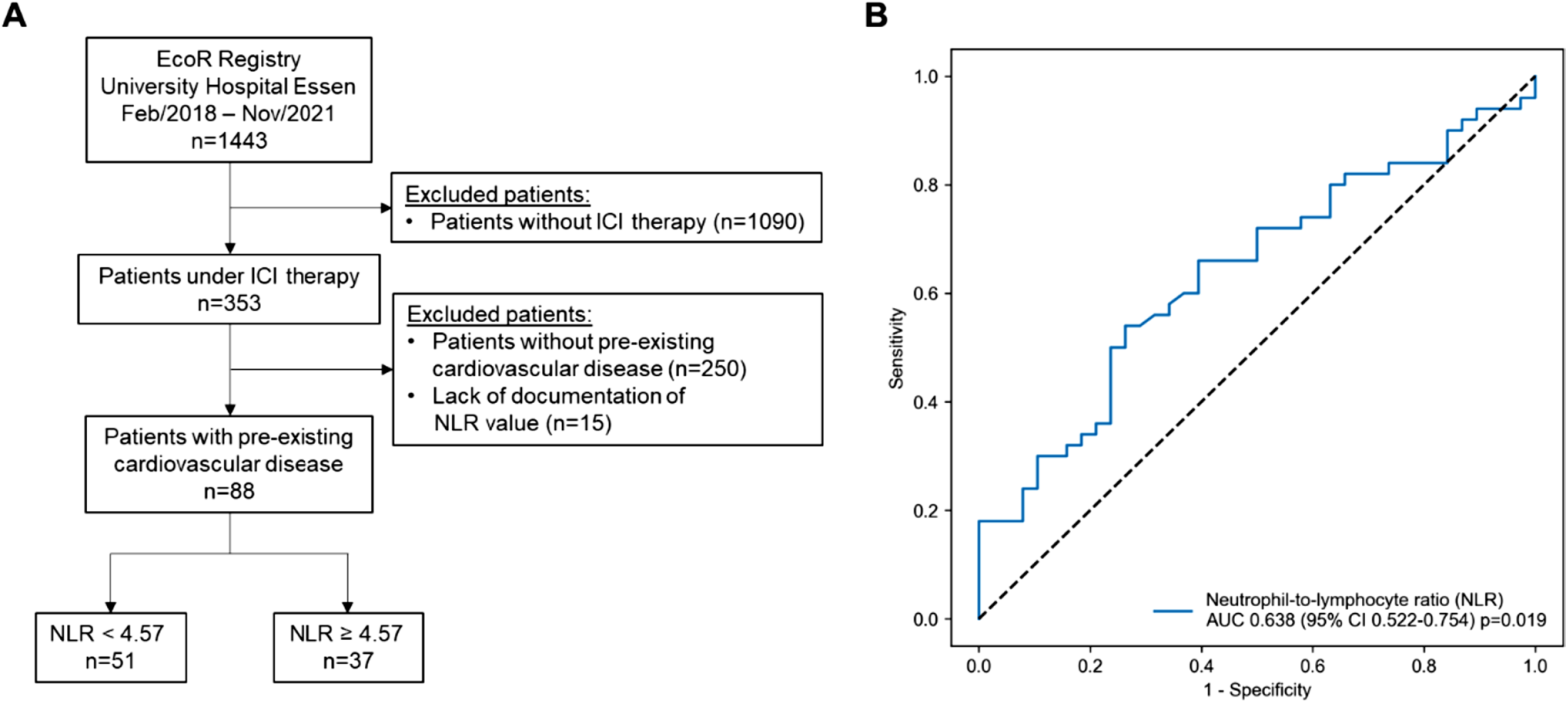
Flowchart of the study population (**A**). Out of 1443 patients form the EcoR registry of the University Hospital Essen, 88 cancer patients under immune checkpoint inhibitor (ICI) therapy with pre-existing cardiovascular disease were identified. The study population was further divided into patients with low (< 4.57) and high (≥ 4.57) neutrophil-to-lymphocyte-ratio (NLR) according to the receiver operator characteristic (ROC) curve analysis of NLR for prediction of cancer therapy-related cardiovascular toxicity (**B**). AUC, area under the curve; CI, confidence interval

The study population was part of the prospective Essen cardio-oncology registry (ECoR). The registry was approved by the local ethics committee (19-8632-BO) and includes all patients attending to the local outpatient cardio-oncology unit, who provide written informed consent. In this study, cancer patients under ICI therapy with pre-existing cardiovascular disease, like coronary artery disease, congestive heart failure, peripheral or cerebral artery occlusive disease, valvular heart disease, atrial fibrillation or history of stroke were included. Exclusion criteria were no ICI therapy, no pre-existing cardiovascular disease and no documentation of NLR values. From 1443 patients, enrolled between July 2018 and November 2021, we analyzed a subgroup of 88 cancer patient with pre-existing cardiovascular disease treated with ICI therapy. Excluded were 1090 patients not receiving ICI therapy, 250 patients without pre-existing cardiovascular disease and 15 patients due to missing NLR values (Fig. 1A). The local cardio-oncology outpatient unit performed patient enrollment and follow-up. Baseline demographics, cardiovascular history, risk factors, cancer treatment and cancer-related characteristics were recorded. Blood samples were obtained and clinical examination, electrocardiography and echocardiography were performed at patient enrollment. NLR values were calculated as a ratio of absolute neutrophil count to absolute lymphocyte count. According to the optimal predictive NLR cut-off value for CTR-CVT determined by receiver operator characteristic (ROC), we divided the study population into two groups (Fig. 1B). During follow-up, CTR-CVT was assessed using clinical examination, electrocardiography, non-invasive cardiac imaging (echocardiography and cardiac magnetic resonance imaging (CMR)) and measurement of cardiac biomarkers (high-sensitive troponin, brain natriuretic peptide (BNP) and N-terminal prohormone of brain natriuretic peptide (NT-proBNP)). CTRCD, myocarditis, vascular toxicity, new onset of arterial hypertension and arrhythmia or QTc prolongation were defined as CTR-CVT according to the diagnosis criteria of the European guidelines on cardio-oncology [17]. Vascular toxicity was defined as pulmonary embolism, deep venous thrombosis, myocardial infarction and stroke. Arrhythmia was defined as atrial fibrillation and sinus tachycardia besides QTc prolongation. Myocarditis was diagnosed as clinical diagnosis consisting of relevant troponin elevation in combination with positive CMR diagnostic for acute myocarditis and exclusion of CAD progression. The median follow-up was 357 days (interquartile range (IQR): 150–509 days).

### Statistical analysis

Categorical variables are given as absolute and relative frequencies (%). Association between these variables was evaluated by the use of chi-squared test. Distribution of continuous variables was assessed using Kolmogorov–Smirnov test. Normally distributed continuous variables were expressed as mean ± standard deviation (SD), and Student’s *t* test was used for comparison. In case of non-normal distribution, continuous variables were displayed as median with IQR. These variables were compared using Mann–Whitney *U* test. ROC curve analysis was performed for cut-off calculation, and the optimal NLR cut-off value was determined using Youden’s index. The predictive capacity of NLR for overall CTR-CVT was also compared to NT-proBNP and high-sensitive troponin by means of the area under the ROC curve (AUC). Kaplan–Meier cumulative event curves showed overall CTR-CVT in dependence of low and high NLR values and were compared using log-rank test. Patients lost to follow-up were treated as censored observations. Median follow-up time was calculated by the reverse Kaplan–Meier method. Skewed data were logarithmically transformed to improve model stability. The association between logarithmized NLR values (log2(NLR)) and CTR-CVT was examined using an univariable Cox regression. Additionally, different multivariable Cox regression models were computed to show the adjusted association of logarithmized NLR values and overall CTR-CVT. The significance level was set as *p < *0.05. All statistical analyses were performed using IBM SPSS 29.0 software (SPSS Inc.). Figures of ROC curve analysis and Kaplan–Meier cumulative event curves were generated with custom Python scripts using appropriate libraries.

## Results

In our study population of 88 patients (mean age ± SD = 69 ± 11 years; 25% female; Table 1), melanoma (51.1%) was the most prevalent cancer entity (Table 1). CAD (39.8%) and atrial fibrillation (32.9%) were the most frequent cardiovascular comorbidities (Table 1). NLR values were assessed at time of patient enrollment at the local cardio-oncology outpatient unit. ROC curve analysis with NLR for predicting CTR-CVT resulted in an area under the curve (AUC) of 0.638 (95% confidence interval (CI): 0.522–0.754; *p* = 0.019; Fig. 1b). This analysis revealed an optimum cut-off value of ≥ 4.57 with 54% sensitivity and 74% specificity. Accordingly, 37 patients (42%) presented with a high NLR value of ≥ 4.57 at time of enrollment. Further, ROC curve analysis illustrated that AUC of NLR is a stronger indicator for overall CTR-CVT than NT-proBNP, but inferior compared to high-sensitive troponin in our study cohort (Supplementary Fig. 1A). Addition of NLR to a model containing NT-proBNP and high-sensitive troponin led to an increase in AUC from 0.619 (NT-proBNP + high-sensitive troponin) to 0.706 (NT-proBNP + high-sensitive troponin + NLR; Supplementary Fig. 1B). Clinical baseline characteristics of the study population according to low and high NLR values are presented in Tables 1 and 2. In the group with low NLR values, more patients suffered from melanoma (31 patients vs. 14 patients; *p* = 0.034). Despite a significant higher portion of patients with hypertension in the group with low NLR values (44 patients vs. 24 patients; *p* = 0.018), no differences in co-morbidities between patients with low and high NLR values were observed in this study population. No significant differences in cardiovascular medication were noticeable (Table 2). Concerning cancer manifestation, 61 patients (69.3%) of the study population suffered from metastatic cancer (Table 1). Prior cancer treatment did not differ between patients with low and high NLR values, besides a higher proportion of radiation therapy in patients with high NLR values (10 patients vs. 15 patients; *p* = 0.032; Table 1). There was no difference in the rate of thoracic radiation therapy. In patients receiving ICI therapy before patient enrollment (61.4%), a new therapy cycle was initiated with a medium of 35 days before patient enrollment. ICI therapy was started in naïve patients with a median of 4 days before patient enrollment. Most of the patients received PD-1 inhibitors as ICI therapy (78 patients, 88.6%) with 48 patients (54.5%) treated with nivolumab and 27 patients (30.7%) with pembrolizumab (Table 2). 17 patients (19.3%) obtained PDL-1 inhibitors with avelumab in the majority (10 patients, 11.4%) and 28 patients (31.8%) received the CTLA-4 inhibitor ipilimumab. More patients with low NLR values were treated with a combination of ipilimumab and a PD-1 inhibitor (20 patients vs. 7 patients; *p* = 0.042).

**Table 1:** Baseline characteristics

	Total (*n = *88)	NLR < 4.57 (*n = *51)	NLR ≥ 4.57 (*n = *37)	*p* value
Age (years), mean ± SD	69 ± 11	69 ± 12	70 ± 11	0.582
Sex (female), *n* (%)	22 (25.0)	15 (29.4)	7 (18.9)	0.262
BMI, median (IQR)	26.6 (24.2–30.6)	27.4 (24.8–30.8)	25.5 (23.5–29.4)	0.067
Cancer disease, *n* (%)				
Melanoma	45 (51.1)	31 (60.8)	14 (37.8)	0.034*
Merkel cell carcinoma	10 (11.4)	5 (9.8)	5 (13.5)	0.588
Cutaneous squamous cell carcinoma	13 (14.8)	5 (9.8)	8 (21.6)	0.123
NSCLC	12 (13.6)	6 (11.8)	6 (16.2)	0.548
Other	13 (14.8)	6 (11.8)	7 (18.9)	0.350
Metastasis	61 (69.3)	34 (66.7)	27 (73.0)	0.828
≥ 2 cancer entities (in the last 5 years)	20 (22.7)	13 (25.5)	7 (18.9)	0.468
Cancer progression	6 (6.8)	4 (7.8)	2 (5.4)	0.601
Cancer recurrence	22 (25.0)	16 (31.4)	6 (16.2)	0.108
Prior cancer treatment, *n* (%)				
Surgery	73 (83.0)	42 (82.4)	31 (83.8)	0.860
Radiation	25 (28.4)	10 (19.6)	15 (40.5)	0.032*
Thoracic radiation	9 (10.2)	5 (9.8)	4 (10.8)	0.902
Chemotherapy	19 (21.6)	8 (15.7)	11 (29.7)	0.114
Anthracycline	2 (2.3)	0	2 (5.4)	0.093
Platinum-based chemotherapy	17 (19.3)	7 (13.7)	10 (27.0)	0.119
Other chemotherapy	4 (4.5)	1 (2.0)	3 (8.1)	0.172
Tyrosine kinase inhibitor	5 (5.7)	2 (3.9)	3 (8.1)	0.402
MEK inhibitor	7 (8.0)	4 (7.8)	3 (8.1)	0.964
B-Raf inhibitor	6 (6.8)	4 (7.8)	2 (5.4)	0.654
Taxane	8 (9.1)	4 (7.8)	4 (10.8)	0.633
Prior ICI therapy	54 (61.4)	28 (54.9)	26 (70.3)	0.144
Cardiovascular comorbidities, *n* (%)				
Coronary artery disease	35 (39.8)	20 (39.2)	15 (40.5)	0.900
Previous PCI	21 (23.9)	14 (27.5)	7 (18.9)	0.354
CABG	13 (14.8)	9 (17.6)	4 (10.8)	0.372
Previous myocardial infarction	12 (13.6)	7 (13.7)	5 (13.5)	0.977
Congestive heart failure	15 (17.0)	8 (15.7)	7 (18.9)	0.691
Peripheral artery occlusive disease	9 (10.2)	4 (7.8)	5 (13.5)	0.386
Cerebral artery occlusive disease	8 (9.1)	5 (9.8)	3 (8.1)	0.785
History of stroke	20 (22.7)	14 (27.4)	6 (16.2)	0.214
Valvular heart disease	5 (5.7)	3 (5.9)	2 (5.4)	0.836
Atrial fibrillation	29 (32.9)	15 (29.4)	14 (37.8)	0.406
Pacemaker	6 (6.8)	2 (3.9)	4 (10.8)	0.206
Chronic kidney disease	10 (11.4)	7 (13.7)	3 (8.1)	0.412
COPD	7 (8.0)	4 (7.8)	3 (8.1)	0.964
Hypertension	68 (77.3)	44 (86.3)	24 (64.9)	0.018*
Diabetes mellitus	25 (28.4)	18 (35.3)	7 (18.9)	0.093
Dyslipidemia	28 (31.8)	19 (37.3)	9 (24.3)	0.199
History of smoking (current or past)	15 (17.0)	9 (17.6)	6 (16.2)	0.917
Obesity	25 (28.4)	17 (33.3)	8 (21.6)	0.229

Continuous variables are expressed as mean ± standard deviation (SD) or median (interquartile range (IQR)) in case of non-normally distributed data. Categorial variables are shown as frequencies and percentages (%)

*NLR* neutrophil-to-lymphocyte ratio; *BMI* body mass index; *NSCLC* non-small-cell lung cancer; *MEK* mitogen-activated protein kinase kinase; *B-Raf* B-rapidly accelerated fibrosarcoma; *ICI* immune checkpoint inhibitor; *PCI* percutaneous coronary intervention; *CABG* coronary artery bypass graft; *COPD* chronic obstructive pulmonary disease

*Statistically significant difference between cancer patients with low and high NLR

**Table 2:** Cardiovascular medication and ICI therapy

	Total (*n = *88)	NLR < 4.57 (*n = *51)	NLR ≥ 4.57 (*n = *37)	*p* value
Cardiovascular medication, *n* (%)				
ASS	35 (39.8)	22 (43.1)	13 (35.1)	0.463
Statin	36 (40.9)	21 (41.2)	15 (40.5)	0.975
β blocker	55 (62.5)	34 (66.7)	21 (56.8)	0.357
ACEi	32 (36.3)	18 (35.3)	14 (37.8)	0.785
ARB	21 (23.9)	15 (29.4)	6 (16.2)	0.156
MRA	10 (11.4)	5 (9.8)	5 (13.5)	0.579
ARNI	1 (1.1)	1 (2.0)	0	0.393
Digitalis	4 (4.5)	1 (2.0)	3 (8.1)	0.169
Oral anticoagulation	35 (39.8)	17 (33.3)	18 (48.6)	0.321
ICI therapy, *n* (%)				
PD-1	78 (88.6)	44 (86.3)	34 (91.9)	0.412
Nivolumab	48 (54.5)	30 (58.8)	18 (48.6)	0.344
Pembrolizumab	27 (30.7)	17 (33.3)	10 (27.0)	0.527
Cemiplimab	11 (12.5)	3 (5.9)	8 (21.6)	0.028*
Spartalizumab	1 (1.1)	1 (2.0)	0	0.392
PDL-1	17 (19.3)	11 (21.6)	6 (16.2)	0.530
Avelumab	10 (11.4)	6 (11.8)	4 (10.8)	0.889
Durvalumab	2 (2.3)	2 (3.9)	0	0.223
Atezolizumab	5 (5.7)	3 (5.9)	2 (5.4)	0.924
CTLA-4 (Ipilimumab)	28 (31.8)	20 (39.2)	8 (21.6)	0.080
CTLA-4 + PD-1	27 (30.7)	20 (39.2)	7 (18.9)	0.042*
Duration of ICI therapy (days), median (IQR)	143 (48–382)	158 (63–393)	99 (42–368)	0.272

Data shown as frequencies and percentages (%) or median (interquartile range (IQR)) in case of duration of ICI therapy

*NLR* neutrophil-to-lymphocyte ratio; *ASS* acetylsalicylic acid; *ACEi* angiotensin-converting enzyme inhibitor; *ARB* angiotensin receptor blocker; *MRA* mineralocorticoid receptor antagonist; *ARNI* angiotensin receptor neprilysin inhibitor; *PD-1* programmed cell death protein 1; *PDL-1* programmed cell death 1 ligand-1; *CTLA-4* cytotoxic T lymphocyte-associated antigen-4

*Statistically significant difference between cancer patients with low and high NLR

Baseline laboratory values, assessed at patient enrollment, are depicted in Table 3. The median NLR value was 2.7 in patients with low NLR value and 6.5 in patients with high NLR value. Patients with high NLR value showed higher levels of white blood cell count (6.4/nl vs. 7.9/nl; *p* = 0.006). High NLR values were driven by a higher count of neutrophils (4.0/nl vs. 5.8/nl; *p < *0.001) as well as reduction in lymphocyte count (1.5/nl vs. 0.9/nl; *p < *0.001). There were no further significant differences in any laboratory parameters including hemoglobin or inflammatory and cardiac biomarkers between patients with low and high NLR values. Functional cardiac parameters were measured at patient enrollment using electrocardiography (ECG) and echocardiography. Here, no differences were observed between patients with low and high NLR values in this study population (Table 4).

**Table 3:** Baseline laboratory parameters

	Total (*n = *88)	NLR < 4.57 (*n = *51)	NLR ≥ 4.57 (*n = *37)	*p* value
Hemoglobin (g/dl)	12.6 (10.9–14.1)	12.6 (11.2–14.1)	12.5 (10.6–14.2)	0.663
White blood cell count (count/nl)	7.0 (5.6–9.3)	6.4 (5.3–8.3)	7.9 (6.2–11.3)	0.006*
Neutrophils (count/nl)	4.6 (3.7–6.8)	4.0 (3.3–5.1)	5.8 (4.6–8.6)	< 0.001*
Lymphocytes (count/nl)	1.3 (0.9–1.7)	1.5 (1.2–1.9)	0.9 (0.7–1.3)	< 0.001*
NLR	4.2 (2.5–6.2)	2.7 (2.0–3.9)	6.5 (5.4–7.8)	< 0.001*
CRP (mg/dl)	0.7 (0.4–2.3)	0.5 (0.4–1.7)	0.9 (0.4–2.9)	0.224
Monocytes (count/nl)	0.6 (0.5–0.8)	0.6 (0.5–0.8)	0.6 (0.5–0.9)	0.291
Platelets (count/nl)	225.5 (191.3–286.0)	221.0 (191.0–264.0)	232.0 (186.0–316.0)	0.548

Parameters are displayed as median (interquartile range (IQR))

*NLR* neutrophil-to-lymphocyte ratio; *CRP* C-reactive protein

*Statistically significant difference between cancer patients with low and high NLR

**Table 4:** Functional cardiac parameters at baseline

	Total (*n = *88)	NLR < 4.57 (*n = *51)	NLR ≥ 4.57 (*n = *37)	*p* value
ECG parameters				
Heart rate (bpm)	78 ± 16	76 ± 17	81 ± 15	0.191
QRS (ms)	92.0 (83.5–106.5)	94.0 (82.0–106.0)	92.0 (86.0–114.0)	0.530
QTc (ms)	443.6 ± 34.0	443.0 ± 35.8	444.5 ± 31.7	0.851
Echocardiography				
LV-EF (%)	55.2 ± 8.6	56.8 ± 7.2	53.1 ± 10.0	0.059
LV GLS (%)	− 19.7 (− 22.1 to − 16.7)	− 19.8 (− 22.3 to − 17.1)	− 19.6 (− 20.8 to − 13.7)	0.203
LAVI (ml/m^2^)	29.3 ± 12.7	29.5 ± 12.7	29.1 ± 12.8	0.890
*E*/*E*' (ratio)	10.5 ± 3.4	10.2 ± 3.1	10.8 ± 4.0	0.707
sPAP (mmHg)	32.9 ± 10.8	30.8 ± 8.5	35.9 ± 13.1	0.098
TAPSE (mm)	24.3 ± 5.9	24.2 ± 6.0	24.3 ± 5.9	0.975

Variables are expressed as mean ± standard deviation (SD) or median (interquartile range (IQR)) in case of non-normally distributed data

*NLR* neutrophil-to-lymphocyte ratio; *LV-EF* left ventricular ejection fraction; *LV GLS* left ventricular global longitudinal strain; *LAVI* left atrium volume index; *sPAP* systolic pulmonary artery pressure; *TAPSE* tricuspid annular plane systolic excursion

**Fig. 2:**
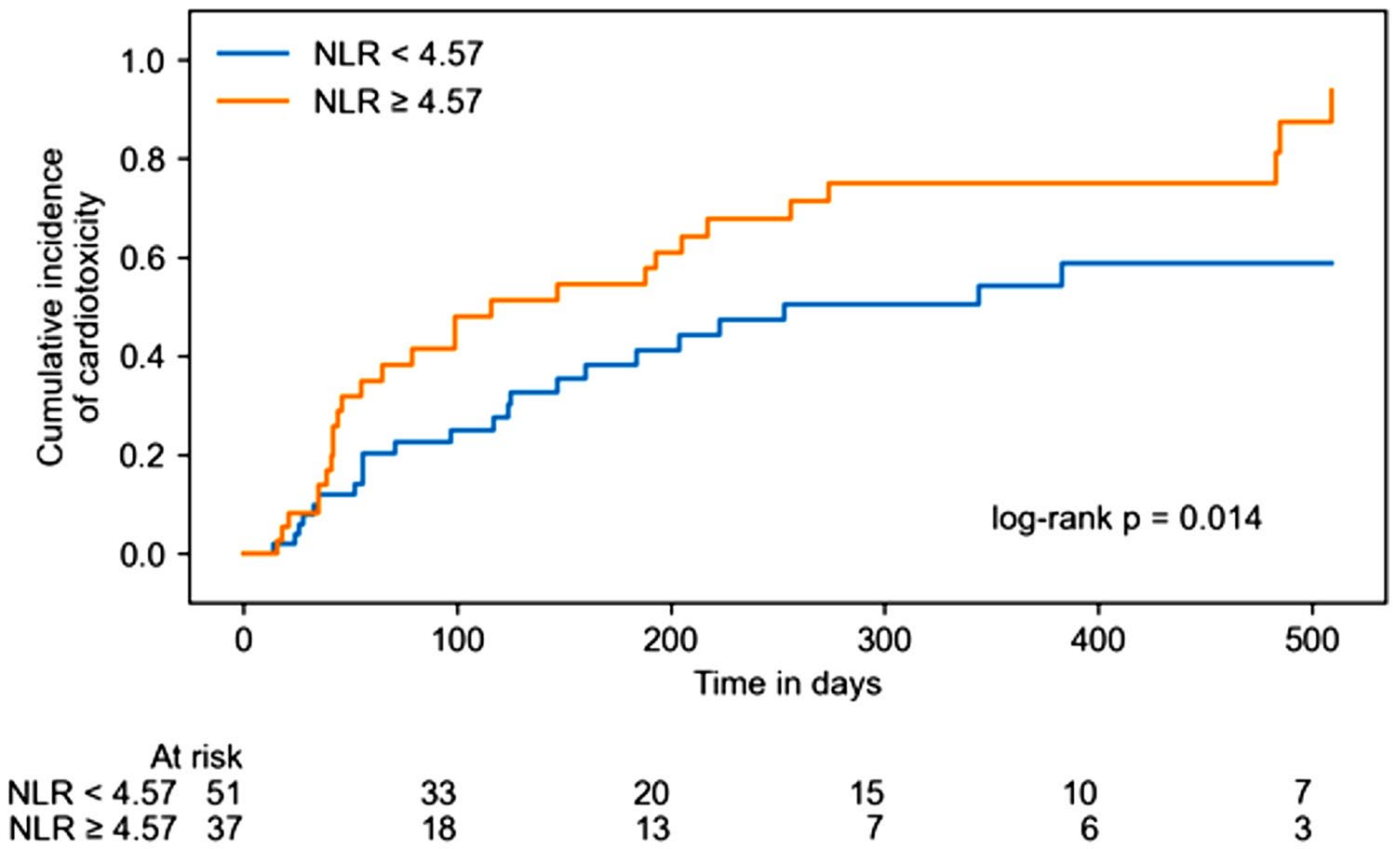
Kaplan–Meier cumulative event curves for overall cancer therapy-related cardiovascular toxicity (CTR-CVT) with patients separated by low (< 4.57) and high (≥ 4.57) neutrophil-to-lymphocyte ratios (NLR)

Overall CTR-CVT, consisting of CTRCD, myocarditis, vascular toxicity, new onset of arterial hypertension and arrhythmia or QTc prolongation, occurred in 50 of the 88 patients (56.8%) (17 patients with CTRCD (19.3%), 4 patients with myocarditis (4.5%), 17 patients with vascular toxicity (19.3%), 3 patients with new onset of arterial hypertension (3.4%) and 22 patients with arrhythmia or QTc prolongation (25.0%); Table 5). 14 patients (15.9%) showed more than one cardiotoxic entity. Univariable Cox regression analysis of logarithmized NLR values at patient enrollment showed a moderate association to increased overall CTR-CVT (hazard ratio (HR): 1.443; 95% CI: 1.082–1.925; *p* = 0.013). This was mainly attributable to a significant association of NLR with myocarditis (HR: 5.556; 95% CI: 2.077–14.861); *p < *0.001) and arrhythmia or QTc prolongation (HR: 1.741; 95% CI: 1.161–2.610; *p* = 0.007). NLR values ≥ 4.57 were significantly associated with overall CTR-CVT (log-rank *p* = 0.014; Fig. 2). The association of logarithmized NLR values with arrhythmia events was mainly referable to a significant association with atrial fibrillation (HR: 2.107; 95% CI: 1.311–3.389; p = 0.002; Supplementary Table 1) and QTc prolongation (HR: 2.260; 95% CI: 1.064–4.802; p = 0.034; Supplementary Table 1). In ICI therapy naïve patients, univariate Cox regression analysis showed a significant association of logarithmized NLR values with overall CTR-CVT (HR: 2.563; 95% CI 1.074–6.116; p = 0.034; Supplementary Table 2).

**Table 5:** Univariable Cox regression for log2(NLR)

Outcome	Number of events	Estimated hazard ratio (95% CI)	*p* value
Overall CTR-CVT	50	1.443 (1.082–1.925)	0.013*
CTRCD	17	1.448 (0.799–2.626)	0.222
Myocarditis	4	5.556 (2.077–14.861)	< 0.001*
Vascular toxicity	17	1.314 (0.794–2.175)	0.288
New onset of arterial hypertension	3	1.994 (0.682–5.826)	0.207
Arrhythmia or QTc prolongation	22	1.741 (1.161–2.610)	0.007*

*NLR* neutrophil-to-lymphocyte ratio; *CI* confidence interval; *CTR-CVT* cancer therapy-related cardiovascular toxicity; *CTRCD* cancer therapy-related cardiovascular dysfunction

*Statistically significant association between NLR and outcome

Several multivariable Cox regression analyses were performed to adjust for different baseline characteristics. The association of NLR with overall CTR-CVT remained significant after adjustment for age and sex (HR: 1.441; 95% CI: 1.069–1.942; *p* = 0.016; Table 6 – multivariable model 1). Similar results were observed after further adjustment for cardiovascular risk factors and comorbidities, including hypertension, dyslipidemia, diabetes mellitus, obesity, history of smoking, CAD, congestive heart failure, valvular heart disease, coronary artery bypass graft (CABG), peripheral and cerebral artery occlusive disease, previous myocardial infarction, percutaneous coronary intervention (PCI) or stroke and atrial fibrillation (HR: 1.464; 95% CI: 1.028–2.084; *p* = 0.034; Table 6, multivariable model 2). However, this significant association was lost after additional adjustment for cancer entities of the examined patients (HR: 1.299; 95% CI: 0.814–2.073; *p* = 0.272; Table 6, multivariable model 3).

**Table 6:** Multivariable Cox Regression for log2(NLR)

Outcome	Model 1		Model 2		Model 3	
	Estimated hazard ratio (95% CI)	*p* value	Estimated hazard ratio (95% CI)	*p* value	Estimated hazard ratio (95% CI)	*p* value
Overall CTR-CVT	1.441 (1.069–1.942)	0.016*	1.464 (1.028–2.084)	0.034*	1.299 (0.814–2.073)	0.272

Model 1: Adjustment for age and sex; Model 2: Adjustment for age, sex, hypertension, dyslipidemia, diabetes mellitus, obesity, history of smoking, coronary artery disease (CAD), congestive heart failure, valvular heart disease, coronary artery bypass graft (CABG), peripheral and cerebral artery occlusive disease, previous myocardial infarction, percutaneous coronary intervention (PCI) or stroke and atrial fibrillation; Model 3: Adjustment for age, sex, hypertension, dyslipidemia, diabetes mellitus, obesity, history of smoking, coronary artery disease (CAD), congestive heart failure, valvular heart disease, coronary artery bypass graft (CABG), peripheral and cerebral artery occlusive disease, previous myocardial infarction, percutaneous coronary intervention (PCI) or stroke, atrial fibrillation and cancer entities

*NLR* neutrophil-to-lymphocyte ratio; *CI* confidence interval; *CTR-CVT* cancer therapy-related cardiovascular toxicity

*Statistically significant association between NLR and outcome

## Discussion

This study examined the association of NLR with CTR-CVT in cancer patients under ICI therapy with pre-existing cardiovascular disease. NLR was significant associated with overall CTR-CVT, mainly driven by the incidence of myocarditis and arrhythmia or QTc prolongation. This association remains after adjustment for age, sex, cardiovascular risk factors and co-morbidities. However, the significance was lost after further adjustment for cancer entities.

Compared to the incidence of CTR-CVT under ICI therapy in general, a relatively high proportion of patients (56.8%) included in our study developed a cardiotoxic event. Several studies mostly reported a CTR-CVT incidence of 6–7% under ICI therapy [35, 36]. These studies mainly defined CTR-CVT as cardiac immune-related adverse events (irAE), including myocarditis, acute coronary syndrome, Takotsubo syndrome and pericardial disease. We investigated the whole spectrum of CTR-CVT, recently defined by the European guidelines on cardio-oncology [17]. Therefore, we additionally included some rather mild diagnosis criteria for CTR-CVT, like cardiac dysfunction, new onset of arterial hypertension or arrhythmia. This might result in a higher incidence of CTR-CVT in our collective. Furthermore, the patients investigated in this study are severe diseased and at higher risk developing CTR-CVT and therefore more likely to attend to our local cardio-oncology outpatient unit, which creates a selection bias. This might explain a higher incidence of myocarditis (4.5%), observed in our study, as other studies reported an incidence of ICI-related myocarditis up to 1% [37, 38]. So far, there are no specific trials estimating the incidence of cardiotoxic events in these high-risk cancer patients with pre-existing cardiovascular disease. Our study population is comparably small; therefore, larger trials are needed to portray a more representative incidence of CTR-CVT in these patients.

Although, sensitivity and specificity of NLR for prediction of CTR-CVT in our study cohort were not ideal, they increased the diagnostic validity of the established parameters like NT-proBNP and high-sensitive troponin, highlighting the additional benefit of this economically biomarker. Also, an AUC of 0.63 appears moderate for the prediction of overall CTR-CVT, but compared to other studies, investigating the association of NLR with cardiotoxicity under anti-cancer treatment similar values for AUC is reported [33]. The same applies to studies examining the association of NLR with mortality or myocardial injury in other non-cancer collectives [39–41].

These results further confirm the association of NLR with CTR-CVT as reported before in other studies. In patients with breast cancer under anthracycline therapy, NLR was associated with deterioration of left ventricular global longitudinal strain (GLS) [33]. High NLR values were also observed in patients with ICI-related myocarditis and arrhythmia [32, 42]. Monitoring of NLR in patients under anti-PD1 therapy was predictive for immune-related adverse events in general [43]. In patients with ICI therapy-related myocarditis, high NLR was also associated with major adverse cardiac events (MACE) [34]. Association of NLR with CTR-CVT was also described in lung cancer patients after initiation of ICI therapy [44]. However, one study showed unaltered NLR in patients developing CTR-CVT under ICI therapy [35]. These patients primarily suffered from respiratory or gastrointestinal tumor disease. The only CTR-CVT endpoint in this study was myocarditis, whereas our study included all entities of CTR-CVT described by the European guidelines on cardio-oncology. In addition, our study population differed considerably, as the majority suffered from melanoma and other skin cancer entities. Apart from cancer patients, there are studies demonstrating a predictive capacity of NLR with cardiovascular injury in a variety of diseases. There is an association of NLR with severity of myocarditis reported [45]. In patients with acute coronary syndrome, NLR showed predictive capacity for myocardial damage and cardiac dysfunction as well as arrhythmia [46, 47]. In other studies, high NLR forecasted the onset of atrial fibrillation [48–50]. Moreover, high NLR values were observed in patients with QT prolongation [51].

High NLR values certainly reflect high inflammatory activity [52]. This is not exclusively observed in patients under ICI therapy and therefore not specific for prediction of CTR-CVT in ICI recipients. Previous studies, however, have concluded that NLR may predict cardiotoxicity in breast cancer patients under anthracycline treatment [33]. In addition, NLR is associated with mortality in cancer patients independent of anti-cancer treatment [53–55]. Furthermore, NLR is a surrogate marker of poor outcomes in the field of cardiovascular disease [27–29]. In our study, NLR is associated with overall CTR-CVT in high-risk cancer patients under ICI therapy highlighting high inflammatory activity as a possible mediator for CTR-CVT in these patients.

Several possible underlying mechanisms are reported for pro-arrhythmic properties of neutrophils in other cardiac disease models. After myocardial infarction, lipocalin-2 (Lcn2)-mediated production of reactive oxygen species (ROS) promotes ventricular arrhythmia [56]. Production of neutrophil extracellular traps (NETs) contributes to progression of atrial fibrillation [57]. Furthermore, the integrin CD11b might be linked to atrial fibrosis and the induction of atrial fibrillation [58]. These mechanisms could be responsible for the association of NLR with arrhythmia and should be subject of further investigations.

High NLR values observed in our study population seemed to be driven by an increase of neutrophils and decrease of lymphocytes. Higher levels of neutrophils reflect a systemic inflammatory status leading to the production of reactive oxygen species and pro-inflammatory cytokines, which can promote myocardial damage [59–62]. In addition, elevated levels of cortisol during a stressed state are associated with a reduction in lymphocyte count, which is accompanied by enhanced tissue injury [63]. These two conditions are able to cause myocardial damage reflecting in an elevated NLR associated with CTR-CVT in our study population.

This study has several limitations. Cardiotoxic confounders like time interval between previous cancer therapies, whether radiation or chemotherapy, were not examined. The same applies for non-cardiovascular premedication apart from reported cancer treatment. Furthermore, our study does not provide any information regarding therapy with corticosteroids as an influencing factor of neutrophil and lymphocyte count in the blood [64, 65]. However, the main portion of included patients suffered from melanoma. For these patients, steroids are mostly used for management of irAE [66]. In our study, only 6.8% of included patients presented with irAE at patient enrollment and could therefore be possibly treated with steroids before study inculsion (Supplementary Table 3). We also analyzed a relatively small number of heterogeneous patients. Therefore, larger prospective clinical trials are needed.

## Conclusion

NLR was associated with overall cancer therapy-related cardiovascular toxicity in cancer patients with pre-existing cardiovascular disease under ICI therapy, representing a high-risk patient collective. The association was mainly driven by the incidence of myocarditis and arrhythmia or QTc prolongation. However, after adjustment for further confounders, this association lost its significance.

### Supplementary Information

Below is the link to the electronic supplementary material.

Supplementary file1 (DOCX 185 KB)

Supplementary file2 (DOCX 13 KB)

Supplementary file3 (DOCX 13 KB)

Supplementary file4 (DOCX 13 KB)

## Data Availability

The dataset analyzed during the current study is available from the corresponding author upon reasonable request.
