# Supplementary material for: High neutrophil-to-lymphocyte ratio is associated with cancer therapy-related cardiovascular toxicity in high-risk cancer patients under immune checkpoint inhibitor therapy
Source: Clin Res Cardiol. 2023 Nov 13;113(2):301–12. doi: 10.1007/s00392-023-02327-9 (PMC10850199; doi:10.1007/s00392-023-02327-9)
Supplement: Supplementary file 1 — Supplementary file1 (DOCX 185 KB) [file 392_2023_2327_MOESM1_ESM.docx]

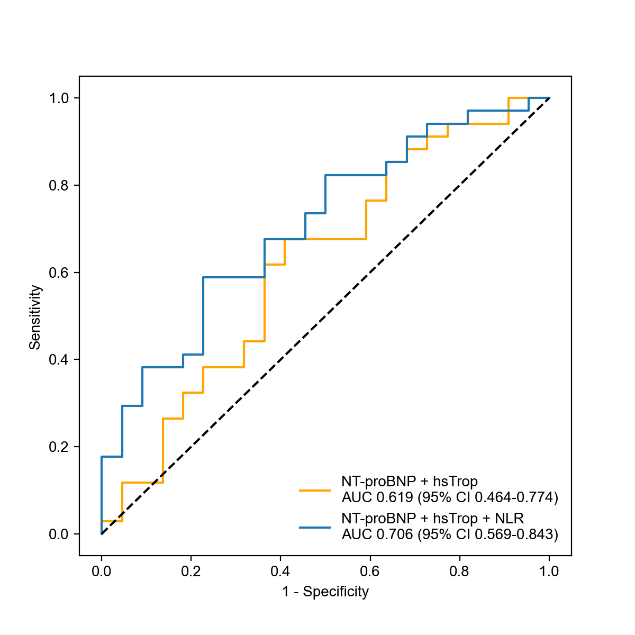

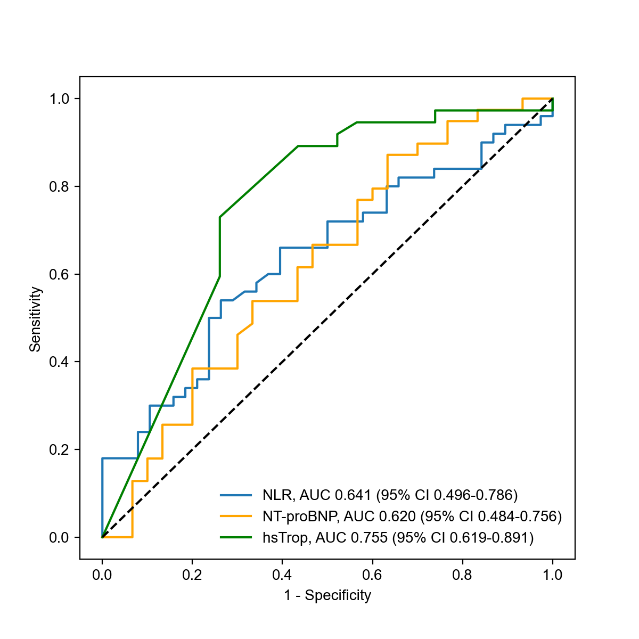


**B**

**A**

**Supplementary Figure 1:** Receiver operator characteristic (ROC) curve analysis of N-terminal prohormone of brain natriuretic peptide (NT-proBNP), high-sensitive troponin (hsTrop) and neutrophil-lymphocyte ratio (NLR) for prediction of overall cancer therapy-related cardiovascular toxicity (CTR-CVT) (A). Comparison of addition of NLR to a model of NT-proBNP and hs-troponin (B). AUC, area under the curve; CI, confidence interval.
