# Supplementary material for: High neutrophil-to-lymphocyte ratio is associated with cancer therapy-related cardiovascular toxicity in high-risk cancer patients under immune checkpoint inhibitor therapy
Source: Clin Res Cardiol. 2023 Nov 13;113(2):301–12. doi: 10.1007/s00392-023-02327-9 (PMC10850199; doi:10.1007/s00392-023-02327-9)
Supplement: Supplementary file 2 — Supplementary file2 (DOCX 13 KB) [file 392_2023_2327_MOESM2_ESM.docx]

**Supplementary Table 1:** Univariable Cox regression for log2(NLR) – subgroups of vascular toxicity and arrhythmia/QTc prolongation

| **Outcome** | **Number of events** | **Estimated hazard ratio (95% CI)** | ***p*-value** |
| --- | --- | --- | --- |
| **Vascular toxicity** |  |  |  |
| Pulmonary embolism | 6 | 1.787 (0.790-4.042) | 0.163 |
| Deep venous thrombosis | 3 | 0.571 (0.119-2.743) | 0.484 |
| Myocardial infarction | 3 | 1.850 (0.647-5.291) | 0.251 |
| Stroke | 5 | 0.342 (0.131-0.893) | 0.028* |
| **Arrhythmia or QTc prolongation** |  |  |  |
| Atrial fibrillation | 14 | 2.107 (1.311-3.389) | 0.002* |
| Sinus tachycardia | 6 | 1.068 (0.449-2.540) | 0.883 |
| QTc prolongation | 6 | 2.260 (1.064-4.802) | 0.034* |

NLR, neutrophil-to-lymphocyte ratio; CI, confidence interval; * statistically significant association between NLR and outcome.
