# Supplementary material for: High neutrophil-to-lymphocyte ratio is associated with cancer therapy-related cardiovascular toxicity in high-risk cancer patients under immune checkpoint inhibitor therapy
Source: Clin Res Cardiol. 2023 Nov 13;113(2):301–12. doi: 10.1007/s00392-023-02327-9 (PMC10850199; doi:10.1007/s00392-023-02327-9)
Supplement: Supplementary file 3 — Supplementary file3 (DOCX 13 KB) [file 392_2023_2327_MOESM3_ESM.docx]

**Supplementary Table 2:** Univariable Cox regression for log2(NLR) – subgroup of ICI therapy naïve patients

| **Outcome** | **Number of events** | **Estimated hazard ratio (95% CI)** | ***p*-value** |
| --- | --- | --- | --- |
| Overall CTR-CVT | 11 | 2.563 (1.074-6.116) | 0.034* |
| CTRCD | 9 | 2.436 (0.953-6.230) | 0.063 |
| Vascular toxicity | 5 | 1.516 (0.435-5.276) | 0.513 |

NLR, neutrophil-to-lymphocyte ratio; CI, confidence interval; CTR-CVT, cancer therapy-related cardiovascular toxicity; CTRCD, cancer therapy-related cardiovascular dysfunction; * statistically significant association between NLR and outcome.
