# Supplementary material for: High neutrophil-to-lymphocyte ratio is associated with cancer therapy-related cardiovascular toxicity in high-risk cancer patients under immune checkpoint inhibitor therapy
Source: Clin Res Cardiol. 2023 Nov 13;113(2):301–12. doi: 10.1007/s00392-023-02327-9 (PMC10850199; doi:10.1007/s00392-023-02327-9)
Supplement: Supplementary file 4 — Supplementary file4 (DOCX 13 KB) [file 392_2023_2327_MOESM4_ESM.docx]

**Supplementary Table 3:** Frequencies of irAE at patient enrollment

|  | **Total**  **(n = 88)** | **NLR < 4.57**  **(n = 51)** | **NLR ≥ 4.57**  **(n = 37)** | ***p*-value** |
| --- | --- | --- | --- | --- |
| irAE, n (%) | 6 (6.8) | 2 (3.9) | 4 (10.8) | 0.206 |
| Pneumonitis, n (%) | 3 (3.4) | 1 (2.0) | 2 (5.4) | 0.379 |
| Hepatitis, n (%) | 3 (3.4) | 1 (2.0) | 2 (5.4) | 0.379 |

Data shown as frequencies and percentages (%). irAE, immune-related adverse events; NLR, neutrophil-to-lymphocyte ratio.
